# Novel Monoclonal Antibody Therapy in a Patient With Treatment-Refractory Warm Autoimmune Hemolytic Anemia

**DOI:** 10.7759/cureus.26051

**Published:** 2022-06-17

**Authors:** Fadi Tahhan, Brandon Huynh, Prissilla Xu

**Affiliations:** 1 Department of Internal Medicine, Adventist Health White Memorial, Los Angeles, USA; 2 Department of Pharmacy Services, Adventist Health White Memorial, Los Angeles, USA

**Keywords:** transfusion requirement, treatment, refractory, sutimlimab-jome, warm autoimmune hemolytic anemia

## Abstract

In autoimmune hemolytic anemia (AIHA), hemolysis is the hallmark symptom. Cold agglutinin disease (CAD) and warm autoimmune hemolytic anemia (wAIHA) are the two major forms of AIHA. Hemolysis is complement dependent in CAD, whereas wAIHA is a partially complement-mediated disorder. At the time of writing, sutimlimab-jome is the only FDA-approved monoclonal antibody in the treatment of CAD. In a case of severe treatment-refractory wAIHA, a two-week course with sutimlimab-jome resulted in a sustained decrease in total bilirubin level and a notable reduction in transfusion requirement, suggesting that it is also effective in the treatment of wAIHA.

## Introduction

Warm autoimmune hemolytic anemia (wAIHA) is the most common form of autoimmune hemolytic anemia (AIHA). It is defined by the presence of autoantibodies that attach to red blood cells at temperatures equal to or greater than normal body temperature (37 degree Celsius), resulting in the destruction of red blood cells by the spleen and/or liver. It is caused by increased erythrocyte destruction by immunoglobulin G (IgG) autoantibodies, with or without complement activation [1]. It is a rare condition, with an estimated incidence of 0.8-3 per 100,000/year in adults, and a prevalence of 17 per 100,000 [2]. Atypical wAIHA and IgG plus complement-mediated disease have a higher treatment failure and higher recurrence rate [3]. In contrast to wAIHA, cold antibody hemolytic anemia is mediated by autoantibodies that cause hemolysis at cold temperatures (typically between 0 and 4 degree Celsius). The hemolytic process in CAD is completely complement mediated [4].

Previously, steroids, immunosuppressants, and splenectomy were the mainstay of AIHA treatment [1]. Plasmapheresis has also been reported in case studies, but its overall effectiveness is uncertain [5]. Recently, novel targeted treatments such as complement-directed therapies are being rapidly developed and explored in AIHA, leading to a changing landscape in the treatment of AIHA and the hope of improving prognosis and outcomes in these rare autoimmune diseases with few therapeutic options. Sutimlimab-jome is the only FDA-approved monoclonal antibody for the treatment of CAD, whereas none has been approved for wAIHA. We report the efficacy of an off-label use of sutimlimab-jome in a case of refractory wAIHA. This case demonstrates that sutimlimab-jome, with its ability to inhibit activation of the classical complement pathway, can offer therapeutic values in complement-mediated wAIHA.

## Case presentation

We present a patient who is a 30-year-old Hispanic female with a past medical history of hypothyroidism and warm antibody autoimmune hemolytic anemia diagnosed three years prior. She presented to the emergency department with complaints of worsening fatigue, dyspnea, dizziness on exertion, headache, chest pain, blurry vision, nausea, vomiting, diarrhea hematuria, sinus tachycardia and anemia with hemoglobin value of 5.1g/dL, and elevated total bilirubin value of 2.5mg/dL. Acute coronary syndrome was ruled out. The patient was started on the standard therapy for wAIHA with steroids and rituximab. A direct antiglobulin test was ordered to confirm autoimmune hemolysis and was found to be positive against IgG, C3b, C3d, and polyspecific antihuman globulin (AHG).

Of note, she was subsequently found to have systemic lupus erythematosus with positive double-stranded DNA (dsDNA), antinuclear antibody (ANA), anticardiolipin, and low C3/C4 after rheumatology workup. Up to hospital day 27, she received a total of 13,000mL (25 units) of packed red blood cells (PRBC) transfusions with fluctuating levels of hemoglobin (5.1-8g/dL) and hematocrit (11.8-24.9g/dL). Initially, the patient was requiring a transfusion approximately every 48-72 hours to maintain a hemoglobin goal of ~7g/dL. Despite several doses of steroids and rituximab, her transfusion requirement began to quickly increase to daily and then 2-3 times per day. Pertinent medication administrations were as follows: 1. Rituximab 375mg/m^2^ on days 1, 7, and 14; 2. Hydroxychloroquine 200mg by mouth (PO) daily from days 12 to 22; 3. Methylprednisolone 250mg IV every 6 hours for three days (days 17-19), then prednisone 40mg daily restarted on day 22; 4. Intravenous immunoglobulin 400mg/kg daily for five days initiated on day 21; 5. Cyclophosphamide 750mg/m^2^ on day 26; and 6. Dexamethasone 40mg IV on days 26-29 and tapered regimen thereafter.

Despite the above treatments, the patient's total bilirubin was steadily rising from 1.3mg/dL to a peak level of 65.3mg/dL. The patient’s condition became increasingly unstable and required transfer to the intensive care unit due to her refractory anemia and tachycardia. At this point in her care, the patient had failed the first- and second-line therapies, and she continued to deteriorate clinically. The patient had failed to respond to rituximab, pulse dose steroids, and intravenous immunoglobulin. Plasmapheresis was considered based on reported successes in a few case studies; however, her hemodynamics did not qualify her for this option. Cyclophosphamide was chosen because of her acute systemic lupus erythematosus flare, its potent immunosuppressive properties, and its efficacy in a case report for steroid-refractory patient similar to ours [6]. Unfortunately, her symptoms and labs did not show significant improvement after cyclophosphamide administration. In discussion with hematologists at a tertiary center, it was recommended to start patient on sutimlimab-jome as last resort in hope of controlling hemolysis, delaying the need for RBC transfusions and stabilizing the patient’s hematologic status to expedite higher level of care transfer. Based on the patient’s body weight, sutimlimab-jome was administered at 6,500mg intravenously weekly for two doses. The patient’s informed consent was obtained due to its off-label use in wAIHA, and the first dose was administered as a two-hour intravenous infusion on day 28 and the second dose on day 35. 

Prior to sutimlimab-jome infusion, red blood cell count was 1,320/dL, hemoglobin value was 3.2g/dL, and hematocrit was 8.5g/dL. The patient was subsequently transfused with two units of PRBCs after the administration of sutimlimab-jome. In the morning after the infusion, red blood cell count increased to 1,890/dL, hemoglobin increased to 6g/dL, and hematocrit increased to 16.9g/dL. Most notably, total bilirubin decreased to 15.2mg/dL and continued to improve in the subsequent days with the lowest level of 7.7mg/dL after the first infusion. This is the first time during the patient's hospitalization that she had a response of more than 1g/dL increase in hemoglobin with only two units of PRBC transfusion. The patient reported global improvement of symptoms during morning rounds. In addition, sutimlimab-jome administration notably decreased transfusion requirement from an average of 2.4 units per day one week before the first dose to 1.8 units per day during the seven days after the first dose. The total bilirubin level further decreased to 4.4mg/dL after the second dose one week later. In a follow-up with the tertiary center, the patient’s transfusion requirements continued to decrease with the improvement in wAIHA markers.

## Discussion

Sutimlimab-jome (Enjaymo®, Sanofi Genzyme Corporation, Cambridge, Massachusetts, USA) is a classical complement inhibitor indicated to decrease the need for red blood cell transfusion due to hemolysis in adults with cold AIHA. It is an IgG subclass 4 (IgG4) humanized monoclonal antibody that inhibits the classical complement pathway and specifically binds to complement protein component 1, s subcomponent (C1s), a serine protease which cleaves C4. Inhibition of the classical complement pathway at the level C1s prevents the deposition of complement opsonins on the surface of red blood cells, resulting in inhibition of hemolysis in patients with cold agglutinin disease (CAD) [7].

The approval of sutimlimab-jome (Enjaymo®) in the United States is based on positive results from the 26-week open-label, single-arm pivotal phase 3 study in patients with CAD (n=24) who have a recent history of blood transfusion, also known as the CARDINAL study. In the study, Enjaymo met its primary efficacy endpoint, which was a composite endpoint defined as the proportion of patients who achieved normalization of hemoglobin (Hgb) level ≥12g/dL or demonstrated an increase from baseline in Hgb level ≥2 g/dL at the treatment assessment time point (mean value from weeks 23, 25, and 26) and no blood transfusion from weeks five through 26 or medications prohibited per the protocol from weeks five through 26. Secondary endpoints were also met, including improvements in hemoglobin and bilirubin [8].

Since its launch in February 2022, there has been no case report describing its use in the treatment of warm AIHA. Our patient remained refractory to conventional treatment with high-dose steroids, intravenous immunoglobulin, cyclophosphamide, rituximab, and high transfusion requirements prior to sutimlimab-jome administration. Both doses resulted in the patient’s self-reported symptomatic improvement as well as improvements in objective clinical findings.

In cold agglutinin disease, hemolysis is thought to be complement dependent, whereas warm AIHA is a partially complement-mediated disorder [9]. If present at a higher titer, production of autoantibodies to IgG may activate complement C in wAIHA [1]. Based on the mechanism of action of sutimlimab-jome, the patient diagnosed with warm AIHA with activation of the classical complement pathway specifically targeted by this inhibitor (C1s) would theoretically be responsive to treatment, despite its sole indication in cold agglutinin diseases.

## Conclusions

This is the first reported case where sutimlimab-jome, an approved treatment solely indicated for CAD, was used in a patient with treatment-refractory wAIHA, resulting in a sustained decrease in total bilirubin and reduced transfusion requirements. It is postulated that our patient had complement-mediated subtype of wAIHA, and activation of the classical complement pathway and its eventual inhibition at the level of C1s by sutimlimab-jome were responsible for this patient’s positive clinical response. Further discussion and investigation are warranted regarding the use of sutimlimab-jome in the treatment of atypical wAIHA with complement activation, despite its sole indication in cold agglutinin disease. This would offer an additional therapeutic opportunity for relapsed and refractory wAIHA, which has remained a unique challenge with few other therapeutic options. Further research is also needed to substantiate the efficacy and safety of sutimlimab-jome in the treatment of wAIHA.
